# About Specialists and Specialism

**Published:** 1889-09-28

**Authors:** 


### Within the Wards.

### ABOUT SPECIALISTS AND SPECIALISM.

It is a very common thing to hear specialists praised; it is also very common to hear them banned and relegated to uncomfortable places. That there is a tendency to too diffuse a specialism few will deny; that specialism is altogether unnecessary and injurious none will affirm. There is no doubt that, within reasonable limits, specialists and specialism are to be encouraged; but it is equally to be hoped that the "Great Toe Hospital," protested against by Sir Andrew Clark, though not created by his fertile imagination, will not pass from the "name" stage to that of a "local habitation" and a subscription list. Mr. Henry J. Butlin, F.R.C.S., gave an address at the recent meeting of the British Medical Association at Leeds, which, though nominally on "Laryngology," was really on the general topic of specialism. Mr. Butlin occupies an unique position with regard to the question, inasmuch as he is, like Sir James Paget, a surgeon in the broad sense of the word, and also a specialist in throat diseases, and head of the department of laryngology at St. Bartholomew's Hospital. That is quite the kind of combination which is most to be desired. If specialism is to develop its highest possibilities, it must be in direct association with general surgery and general medicine. A mere specialist who cares for nothing beyond his specialty, "you know," is self-convicted a fool.

Mr. Butlin's views, it seems to us, are so practical, and so apt for the needs of the moment, both for medical men and the outside world, that we shall confer a distinct advantage on all classes of our readers by quoting them freely. Speaking of the relative importance of specialties in medical practice, he says: "In estimating the value and importance of any specialty regard must be had to the nature and frequency of the more serious diseases to which the special part of the body is subject; to the frequency and discomfort due to minor maladies; to the difficulty of acquiring skill in the use of various instruments required for diagnosis and treatment; to the relief which the specialty is capable of affording; and last, but not least, to the aid which it can render to general medicine and surgery." That puts the whole question into a nutshell. Are there a large number of important diseases attacking any part of the body, the treatment of which is so difficult to learn and to practice that the ordinary practitioner is very likely to be deficiently skilled in it? Then a specialty with regard to that part and those diseases is not only justifiable but indispensable, and that in the interests of medical men as well as of patients.

But, now, if specialties in medicine be really necessary within certain well-defined limits, it is of first-rate importance to the public that a sufficient number of men be found able and willing to learn them, and that adequate facilities be provided for teaching. For, after all, the public must bear in mind that medical knowledge and skill are acquired for their benefit, and that such medical knowledge and skill will only be acquired in response to a general demand—a demand which shall be financially profitable to the doctor. English farmers used to grow enormous quantities of very fine wheat. Why? Because it was their custom and their pleasure partly; but still more because it paid to grow wheat. It took them years and years to give up growing that particular cereal; but since the steady and persistent fall in prices which has obtained during the last few years has had time to operate on their minds, they have ceased to a large extent to produce it. It does not pay. Now medical men love knowledge and skill, but the majority of them, if they do not love money better, at least find money indispensable; and they will no more continue to acquire knowledge and to cultivate skill which does not "pay," than the farmer will continue to grow wheat. We have interpolated this little exordium in order that Mr. Butlin's words on the subject may receive the full measure of consideration to which they are entitled by their importance to the public. "During the past twenty years," he says, "the ordinary pass examinations for licenses and degrees have been made much more difficult. The requirements of the various boards and bodies have been much more severe in all the ordinary subjects of medical education. Students are compelled to spend a longer period in study, and a longer period means a greater sum of money. The time and money thus spent must, in the case of the large majority of students, be devoted to the learning of the foundation subjects, anatomy, physiology, and the like, and to the three great clinical subjects, medicine, surgery, and midwifery. For the study of the various specialties a longer course would be required, and a large number of students cannot afford even an extra three months, which might be devoted to specialties. For," and this is highly important to the public who employ doctors, "the longer time and larger sum of money which have been already devoted to medical education have not thus far been repaid by larger incomes gained by medical men. *On the contrary, the incomes of a large number of medical men are decidedly smaller than they were a few years ago.* . . . . There is a distinct tendency on the part of the public to betake themselves to provident dispensaries, and to get their doctoring done on a cheap contract by combination, apparently caring little what the quality of the doctoring is, provided only it be cheap. That provident dispensaries are or may be a great boon to the working and artisan classes I cannot doubt; but they are taken advantage of by many persons whose position and income are such that one wonders they care to place their health in the hands of some of the men who are in charge of some of these co-operative establishments."

The fact remains that there is a disposition at present to save money in doctor's bills; and under these circumstances, there cannot be a strong desire on the part of students and medical men to spend more money in education.

It is perfectly obvious that special instruction and skill are needed in many departments. It is equally obvious that if a strictly limited few only can afford to study specialties, the value of the specialty will rise, until it will be prohibitory for all but the richest. If specialists are to be educated in sufficient numbers to be within the reach of all, the public must be more liberal towards the profession as a whole, and thus encourage the average student to study specialties in addition to the ordinary curriculum. It is only thus that the best science and skill of the times can be made universally available.

